# Predicted reproductive longevity and women’s facial attractiveness

**DOI:** 10.1371/journal.pone.0248344

**Published:** 2021-03-10

**Authors:** Agnieszka Żelaźniewicz, Judyta Nowak-Kornicka, Klaudia Zbyrowska, Bogusław Pawłowski

**Affiliations:** Department of Human Biology, University of Wrocław, Wrocław, Poland; University of St Andrews, UNITED KINGDOM

## Abstract

Physical attractiveness has been shown to reflect women’s current fecundity level, allowing a man to choose a potentially more fertile partner in mate choice context. However, women vary not only in terms of fecundity level at reproductive age but also in reproductive longevity, both influencing a couple’s long-term reproductive success. Thus, men should choose their potential partner not only based on cues of current fecundity but also on cues of reproductive longevity, and both may be reflected in women’s appearance. In this study, we investigated if a woman’s facial attractiveness at reproductive age reflects anti-Müllerian hormone (AMH) level, a hormone predictor of age at menopause, similarly as it reflects current fecundity level, estimated with estradiol level (E2). Face photographs of 183 healthy women (M_age_ = 28.49, SD_age_ = 2.38), recruited between 2^nd^ - 4^th^ day of the menstrual cycle, were assessed by men in terms of attractiveness. Women’s health status was evaluated based on C-reactive protein level and biochemical blood test. Serum AMH and E2 were measured. The results showed that facial attractiveness was negatively correlated with AMH level, a hormone indicator of expected age at menopause, and positively with E2, indicator of current fecundity level, also when controlled for potential covariates (testosterone, BMI, age). This might result from biological trade-off between high fecundity and the length of reproductive lifespan in women and greater adaptive importance of high fecundity at reproductive age compared to the length of reproductive lifespan.

## Introduction

According to some hypotheses of the adaptive significance of physical attractiveness, traits perceived as attractive are expected to be related with various aspects of an individual’s biological condition [1]. Some previous studies have shown that attractiveness in women is positively correlated with fecundity level, estimated both with estradiol level, the key hormone for predicting successful conception [2], or the number of biological children [3–7]. As women differ in fecundity depending on genetic, developmental, health, and environmental factors [8–10], choosing more attractive (i.e. more fecund) woman in mate choice would be adaptive and might potentially increase male reproductive success. However, women vary not only in terms of fecundity at reproductive age, but also in terms of length of reproductive lifespan (residual time to menopause), an important factor for a couples’ total reproductive success [11]. In humans, inclined towards long-term monogamous pair-bonding [12], this may be especially important due to age-related decrease in fertility, ending in menopause, associated with the ultimate cessation of reproductive functions. Thus, for long-term relationships, where the number of offspring produced by a couple will depend in part on time to a woman’s menopause, men should choose their potential partners, not only based on cues of high current fecundity, but also based on cues of high residual reproductive value [13].

If a woman’s age at natural menopause is important for her partner’s reproductive success, and if age at menopause is linked to some morphological traits, then selective pressure could shape men’s preferences toward morphological traits indicating longer residual time to menopause. The first predictor of high residual reproductive value is young age. Many studies have shown that cues of youth are among the most important determinants of women’s physical attractiveness [14], that, when assessed by men, declines with age [15]. Then, for a given age, the crucial determinant of a woman’s length of reproductive lifespan is predicted age at menopause and preceding time of subfertility, what may vary both across and within populations [11,16–19]. If predicted age at menopause is reflected in morphological cues of women at reproductive age, these traits should influence female attractiveness. As proposed by Bovet et al. [16], it is possible, that morphological cues of current fecundity level (e.g. traits related to estradiol level) may also inform on a woman’s residual reproductive value. Fecundity level within reproductive age and the length of reproductive lifespan may be positively correlated due to common underlying factors, determining a woman’s general biological condition [16], or negatively correlated as a result of a biological trade-off [20]. Alternatively, estimated age at menopause may be independent of cues of current fecundity, and thus may be correlated with different morphological cues or may not be “advertised” at all [16].

So far, only one study has investigated the extent to which face attractiveness of women at reproductive age provides cues to their likely age at menopause. The results showed that women, whose mothers had menopause at older age, are perceived as more attractive [16]. However, although mother’s and daughter’s age of menopause are positively related [21,22], the correlation is not very strong, ranging from 0.22 [21,23] to 0.27 [24]. This correlation seem to be stronger in mother-daughter dyads, characterized by premature menopause [22], but this may be caused by some heritable reproductive disorder, underlying part of the relationship. What is more, menopause defined as the last menstruation, indicating the moment of permanent cease of ovarian function, is preceded by a gradual loss of fertility, dictated by the process of ovarian follicle depletion leading to loss of oocyte quantity and quality [25]. There is a fixed time-interval between the beginning of accelerated decline in fertility and age of menopause, related with lower antral follicle count and asymptomatic onset of subfertility, which also varies between women [26–28]. The age at which a woman becomes subfertile should be also important in mate choice context, and may not be reflected in maternal age at menopause, but can be estimated with hormonal indicators of ovarian ageing.

One of the key hormonal predictors of ovarian ageing and time to menopause (TTM) in younger women is anti-Müllerian hormone (AMH) level [29,30]. AMH is produced by granulosa cells of ovarian follicles during the early stages of follicle development. Its expression starts in primary follicles as soon as recruitment from the primordial follicle pool is initiated, until the early antral stage, during which expression is the strongest, and ceases in follicles with a diameter between 8 and 10 mm [29]. Due to this expression pattern, serum AMH was suggested to reflect a number of early growing follicles, which correlates with a number of primordial follicle and antral follicle count in women, thus constitutes a proxy for the size of a woman’s ovarian reserve [31–33]. After an initial increase until early adulthood, AMH concentration slowly decreases with increasing age, reflecting the decreasing number of oocytes in ovaries, until becoming undetectable c.a. 5 years before menopause, when the stock of primordial follicles is nearly exhausted [30,34–36]. Initial size of the follicle pool and pace of its depletion are highly variable among women, what is reflected in differences of age at menopause. Accordingly, AMH levels may vary significantly in women of the same chronological age, allowing to predict the remaining length of a woman’s reproductive lifespan [29]. As variation in AMH level reflects the quantity of remaining primordial follicles, the range in reproductive capacity, and age of menopause, it indicates a woman’s residual reproductive value more accurately than chronological age [19,29,35]. Women with AMH level at the upper limit of the normal range will enter menopause at a later age compared with women with AMH levels at the lower limit of the normal range [37].

The aim of this study was to investigate if AMH level, a hormone predictor of expected age of menopause, is related to facial attractiveness in women within the reproductive age. In order to better understand how residual reproductive value is related with fecundity level in the reproductive age (verify if the two traits are correlated or traded-off) estradiol level (E2) was measured. E2 is a hormone indicator of current fecundity [2], and also may contribute to perceived women’s facial attractiveness [6]. Furthermore, as women’s facial attractiveness has been shown to be negatively related with testosterone level [38] we controlled for testosterone in the analysis. This is also important as AMH level may be abnormally high in women with polycystic ovary syndrome (PCOS), a condition related with increased testosterone level [39]. Although the study sample included only healthy participants, without fertility and hormonal problems, including testosterone level in statistical analysis allows to control for women who have both relatively high AMH and testosterone levels, what might be related with undiagnosed, emerging PCOS and might influence the studied relationship between AMH and perceived attractiveness.

## Materials and method

This study was a part of a broader project on women’s health, which included 211 participants (M_age_ = 28.51, SD_age_ = 2.37), recruited via social networks, information in local newspapers or on a local radio. All participants were of European descent. The research was approved by the by Ethics Committee of Lower Silesian Chamber of Physicians (2/BO/2016). The general purpose of the study was explained and written consent was obtained for participation in the study and use of data for scientific purposes from all participants. All medical procedures, including participants examination and blood collection, have been conducted by certified medical staff at the medical clinic. All procedure was consistent with the guideline included in the "Declaration of Helsinki–Ethical Principles for Medical Research Involving Human Subjects" formulated by World Medical Association in 2013 (https://www.wma.net/policies-post/wma-declaration-of-helsinki-ethical-principles-for-medical-research-involving-human-subjects/).

### Participants and general procedure

Participants were included in the study sample if they met the following criteria: had no fertility problems (including PCOS), were nulliparous, did not use hormonal contraception or medication, had no chronic disease (diabetes, thyroid problems), and no ongoing health problems (cold, flu, tooth extraction, fever, etc.). Participants were invited to participate in the study between the 2^nd^ and the 4^th^ day of menstrual cycle.

The first step of the study was medical qualification to participate in the study. After this, in order to verify participants’ general health status, biochemical blood tests were performed (morphology with smear and C-reactive protein level evaluation). Fasting blood sample was collected for further hormonal analyses. Height and weight were measured and BMI was calculated in order to control for a potential impact of body adiposity on hormonal profiles or face attractiveness. Face photographs were taken and women answered personal questionnaires that included questions on date of birth, age at menarche, past and current health problems (including hormonal disorders).

Based on collected data, 28 participants were excluded from the analyses due to following reasons: 1) missing face photograph or hormonal analysis (N = 9); 2) inadequate day of menstrual cycle (N = 5); 3) chronic diseases (N = 6); 4) AMH > 20 ng/ml or AMH < 0.5 ng/ml, what may indicate undiagnosed reproductive disorders (N = 4); 5) CRP>10 μg/ml, indicating an ongoing inflammatory state (N = 4). The final analyses included 183 women between 25 and 34 years (M_age_ = 28.49, SD_age_ = 2.38).

### Stimuli and on-line survey

Face photographs were taken with digital still camera (Nikon D7100 with Tamron SP AF 17-50mm F/2.8 XR Di II LD IF camera lens) at a distance of 2.0 m, using the same general settings and constant lighting conditions. Women had no make-up, were asked to pose with a neutral facial expression, to remove glasses or earrings, and to wear a hairband (to make sure that all the face was visible). The background was replaced with a uniform white colour. Ovals were placed around the women’s faces to obscure information about hairstyles.

Photographs were randomly divided into fourteen surveys and each survey contained up to 15 photographs in order to avoid presenting too many stimuli to one responder. Photographs were presented in random order. Men were asked to evaluate women’s facial attractiveness on the scale from 1 (not at all attractive) to 9 (very attractive). Each photo was rated by 100 healthy men of European descent, aged 18–39 years (M_age_ = 23.74, SD_age_ = 4.26), recruited on-line, via social media, internet forums, and university webpages. Inter-rater agreement for attractiveness assessment was high (Cronbach’s α = 0.98) and mean attractiveness ratings were used in the statistical analyses.

### Hormone levels analyses

AMH, total testosterone (tT) and estradiol (E2) levels were evaluated in serum. Fasting blood samples were collected into EDTA Vacutainers (BD®). Serum was separated by centrifugation, then portioned into micro-tubes and stored at -79°C until analyses.

The quantitative measurements of serum anti-Müllerian hormone levels were determined by enzyme-linked immunosorbent assay kit (AMH Gen II ELISA, Beckman Coulter®), with the assay sensitivity of 0.08 mg/mL. All samples and standards were assayed in duplicate and processed according to the manual, supplied with the kit. Intra-assay coefficient of variation was < 5.4%, and inter-assay coefficient of variation was < 7.7%. The absorbance value was measured at 450nm on ASYS UVM 340 (Biochrom®) microplate reader. Standard curve was created by plotting absorbance values for standards (Y axis) against its concentration (X axis). AMH concentration was calculated in relation to the standard curve and expressed in ng/mL.

Quantitative determination of serum total testosterone (tT) concentration was evaluated by enzyme-linked immunoassay using commercial kit (DEMEDITEC, cat. no. DE1559). Inter- and intra-assay coefficients of variation, provided by the manufacturer, were < 10% and < 4.2% respectively, with a test sensitivity 0.08 ng/mL. Standards, controls and serum samples were assayed in duplicate in accordance to manufacturer’s instructions. Absorbance was measured at 450nm on microplate reader (ASYS UVM340, Biochrom®). Hormone concentrations in participants’ serum were calculated in relation to standard curve and expressed in pg/ml.

Estradiol level was measured in certified analytical laboratory (DIAGNOSTYKA®) using Roche’s technology (ElectroChemiLuminescence methods) and measured on Cobas analyzer (Roche Diagnostic). Serum E2 concentration were expressed in pg/mL.

### Statistical methods

As values of AMH, E2 and tT levels were not distributed normally across participants, logarithmic values were used in the analysis.

Although facial appearance ratings were nested in the surveys (the photos were divided between the surveys and rated by different sets of men), preliminary analyses (nested Mixed Model), showed the Intraclass Correlation Coefficient to be equal to 0, indicating that data were not hierarchical, and that unbiased estimates can be obtained from simple correlational analysis and multiple regression.

First, we ran simple correlation analysis between AMH, E2, tT and ratings of face attractiveness. Then we ran regression analysis with perceived face attractiveness as dependent variable and E2, tT, chronological age, and BMI as controlled variables. Analyses were performed with Statistica 12.0 software. The results were interpreted as statistically significant if *p* < 0.05.

## Results

### Descriptive statistics

Mean values, ranges, and standard deviations of facial attractiveness assessments, AMH, E2, tT, and potential covariates (age, age at menarche, and BMI) are presented in Table 1. We found no relationship between age at menarche and AMH level (r = -0.01; *p* = 0.87; 95% CI [-0.15; 0.13]) or facial attractiveness (r = 0.04; *p* = 0.55; 95%CI [-0.10; 0.18]), thus we did not control for age at menarche in the analyses. We found no correlation between BMI and AMH level (r = 0.02; *p* = 0.78; 95%CI [-0.12; 0.16]) and a negative correlation between BMI and E2 level (r = -0.17; *p* = 0.02; 95%CI [-0.31; -0.02]) and positive with tT (r = 0.12; *p* = 0.06; 95%CI [-0.02; 0.26]). BMI was also negatively correlated with facial attractiveness (r = -0.31; *p* < 0.001; 95%CI [-0.43; -0.17]). AMH was negatively related with E2 level (r = -0.15, *p* = 0.04; 95% CI [-0.29; -0.01]).

**Table 1:** Descriptive statistics of the analysed variables (N = 183).

	M	SD	Min	Max
**Age**	28.49	2.38	25.00	34.00
**Age at menarche**	12.82	1.42	9.50	18.00
**AMH [ng/ml]**	5.31	3.05	0.94	16.47
**Estradiol [pg/ml]**	34.77	17.54	5.00	110.00
**Total testosterone [ng/ml]**	0.53	0.20	0.30	1.85
**Face attractiveness [1–9]**	3.70	0.99	1.47	6.32
**BMI**	22.02	3.31	16.34	35.40

### Facial attractiveness, AMH and estradiol levels

**Fig 1:**
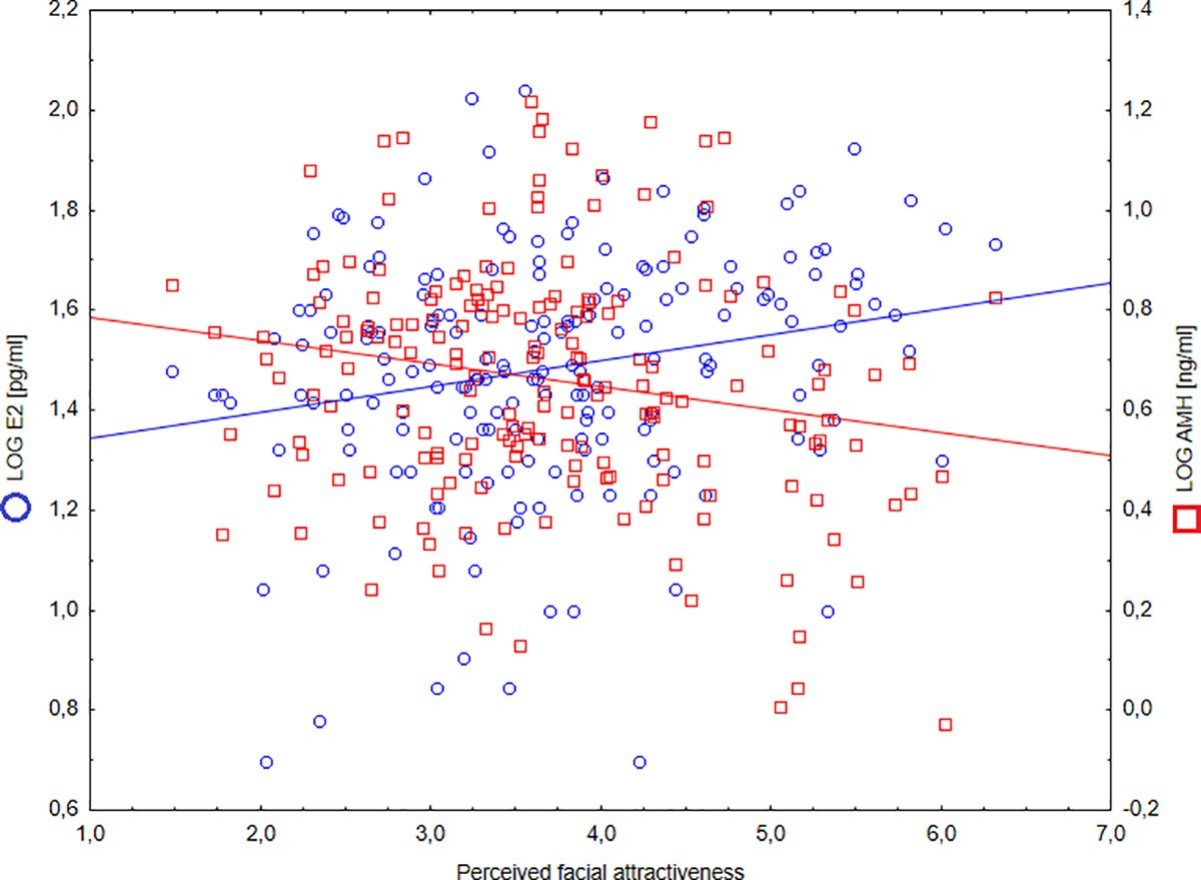
Correlations between AMH and E2 levels and perceived facial attractiveness (N = 183).

The results of the correlation analysis showed that AMH level was negatively related with facial attractiveness (r = -0.19; *p* = 0.01; 95%CI [-0.32; -0.05]). The contrary was observed for the relationship between facial attractiveness and E2 level (r = 0.21; *p* = 0.003; 95%CI [0.07; 0.34]) (Fig 1). Furthermore, tT was negatively related with perceived facial attractiveness (r = -0.15; *p* = 0.049; 95%CI [-0.29; -0.01]).

Regression analysis confirmed that facial attractiveness was negatively related with AMH level and positively correlated with E2 level, also when controlled for BMI, age and tT level (Table 2).

**Table 2:** Results of regression analyses for the relationship between face attractiveness and AMH level, controlled for covariates (N = 183).

	β	SE (β)	t(177)	*p*
**Age**	-0.09	0.07	-1.22	0.22
**LOG AMH**	**-0.15**	**0.07**	**-2.15**	**0.03**
**LOG tT**	-0.09	0.07	-1.28	0.20
**LOG E2**	**0.17**	**0.07**	**2.29**	**0.02**
**BMI**	**-0.27**	**0.07**	**-3.80**	**<0.001**

Bolded values are significant at *p* < 0.05. F(5,177) = 6.89, adj. R^2^ = 0.14, *p* < 0.001).

## Discussion

In contrast to the research hypothesis, the result of this study showed that facial attractiveness of women at reproductive age is negatively related with AMH level. Simultaneously, we found a positive correlation between face attractiveness and estradiol level, a hormone predictor of current fecundity [2], which was also shown in previous studies [6, 40; but see also for negative results 41]. Facial attractiveness was also negatively related with BMI what has been also shown in the previous studies [42,43].

Our results contradict the results obtained by Bovet et al. [16], showing a positive correlation between face attractiveness and predicted length of reproductive lifespan, estimated based on maternal age at menopause. Although, the most recent data on secular trends in age at menopause in Europe are scarce and difficult to compare, there seem to be no major difference between European countries, including Poland and France [44,45], that could explain the contradictory results of the studies. This difference in the study outcomes may be explained by different methods to estimate expected age at menopause employed in the two studies. Although, there is a positive association between mother’s and daughter’s age at menopause, existing estimates of the heritability of menopause age have a wide range [21,22,46]. Also, reported mother’s age at menopause may not be accurate due to the potential risk of recall bias [47]. Furthermore, previous research showed that AMH level is a better predictor of a woman’s TTM, compared to mother’s age at menopause [48,49], due to several reasons. AMH level is influenced by environmental factors that are also related with menopausal age, such as smoking or diet [50,51]. Also, a mother’s age at menopause is determined by genetic factors, that are shared by a mother and a daughter, and by environmental factors acting only on a mother, but not on a daughter [49]. While, a daughter’s age of menopause is influenced both by genetic and environmental factors, with genetic component reflecting not only maternal but also paternal genetic contribution [46,52]. Therefore, whilst information from mother’s age at menopause only reflects the maternal half of genetic influence, AMH level may reflect the sum total of genetic and environmental influences [50], and thus correlates more strongly with actual age at menopause [49]. Additionally, maternal age at menopause may only predict a daughter’s at menopause, whereas women’s fertility decline earlier, what reduce the chance of a successful pregnancy a few years before menopause. The age of the onset of a period of subfertility and infertility that precede menopause differs among women as well [46], and this should be indicated by AMH level (marker of diminishing ovarian reserve) but not by maternal age at menopause.

The results of the study also showed a negative correlation between AMH and E2 levels, what is in line with previous research [53,54]. Experiments in vitro showed that E2 down-regulates AMH expression in primary cultures of human granulosa cells (what in vivo may facilitate reduction of ovarian reserve), and when estradiol concentration reaches a certain threshold, it is capable of completely inhibiting AMH expression through ERβ receptors [55]. This, together with the results of our study, may suggest an existing trade-off between current fecundity, length of reproductive lifespan and a woman’s capability to invest in morphological cues of both. Life-history theory predicts that evolution of fitness-related traits and functions is constrained by the existence of trade-offs between them. Trade-offs are ubiquitous in nature, their existence is explained in the context of resource limitations [56], and may be observed not only between different traits and functions (e.g. immunity and fertility), but also within one function, e.g. different components of reproductive effort. Possibly, there is also a trade-off between high fecundity at reproductive age (the likelihood of fertilization within the cycles at reproductive age) and the length of reproductive lifespan (allowing for reproductive profits in a long-term perspective).

The existence of such trade-off may be confirmed by research showing that older age at menopause is related with using hormonal contraception for longer than a year [57,58; but see for contradictory results: 59,60] and occurrence of irregular cycles before age of 25 [58], which are often anovulatory [61]. Also, some research show that the number of children correlates negatively with AMH level in young women, what may suggest that more fertile women have shorter TTM [62,63]. On the other hand, some research show a positive correlation between AMH level and number of children [64] and that childlessness is linked with younger age at menopause [57,65,66]. However, this correlations may be caused by other variable (e.g. genetic factors or some disease), that causes both low fertility and earlier ovarian failure [66], and thus do not exclude the possible existence of the trade-off between high fecundity at reproductive age and length of reproductive lifespan.

Furthermore, sexual selection may act more strongly on male preferences toward cues of high fecundity at the reproductive age compared to cues of long reproductive lifespan. This presumption might explain the observed finding of a negative relationship between attractiveness and AMH and a simultaneous positive correlation between attractiveness and E2. Firstly, although humans often live in long-term pairbonds, remarriage is common after spousal death and/or divorce, resulting in serial monogamy [67]. Thus, as adult mortality was higher and the expected lifespan was shorter in our evolutionary past [68], men would profit more from mating with highly fecund women compared to mating with women with longer reproductive lifespan. Furthermore, many women (also in traditional societies) give last birth long before the time of menopause, not fully profiting from the length of their reproductive lifespan [69]. Pregnancy in older age is related to a higher risk of pregnancy complications, miscarriage [70], and maternal death [71], what might contribute to an earlier cessation of reproduction [69]. Also, many environmental and life-style factors may impact age at menopause [51,72], influencing the relationship between morphological cues of long reproductive lifespan at younger age and the actual age at menopause. Thus choosing a potential partner based on the cues of current fecundity may bring a greater fitness pay-off, compared to choosing a partner with a potentially long reproductive lifespan.

Finally, some limitations of our study need to be addressed. Both AMH and E2 levels were assessed only at the between-subjects level, based on a single measurement. Although AMH level has been shown to vary across menstrual cycle [73], the extent of variation is small and sampling on any day of the menstrual cycle is expected to adequately reflect ovarian reserve [74]. However, E2 level predicts most reliably a woman’s fecundity if based on repeated sampling across menstrual cycle [75]. Thus, it would be worth to verify the results of our study with repeated AMH and E2 measurements, using longitudinal, rather than cross-sectional design, to assess the relationship between these hormones and a woman’s facial attractiveness.

This is the first study investigating the relationship between AMH level and facial attractiveness in women. The results showed that women perceived as more attractive are characterized by lower AMH, hormonal predictor of age at menopause, and higher E2 levels, hormonal indicator of current fecundity. This might result from biological trade-off between high fecundity and the length of reproductive lifespan in women and greater adaptive importance of high fecundity during reproductive age compared to the length of reproductive lifespan.

## Supporting information

S1 Database (XLSX)
